# Individual prediction of trauma-focused psychotherapy response in youth with posttraumatic stress disorder using resting-state functional connectivity

**DOI:** 10.1016/j.nicl.2021.102898

**Published:** 2021-11-26

**Authors:** Paul Zhutovsky, Jasper B. Zantvoord, Judith B.M. Ensink, Rosanne op den Kelder, Ramon J.L. Lindauer, Guido A. van Wingen

**Affiliations:** a Amsterdam UMC, University of Amsterdam, Department of Psychiatry, Amsterdam Neuroscience, Amsterdam, The Netherlands; b Amsterdam UMC, University of Amsterdam, Department of Child and Adolescent Psychiatry, Amsterdam Neuroscience, Amsterdam, The Netherlands; c Levvel, Academic Centre for Child and Adolescent Psychiatry, Amsterdam, The Netherlands; d Research Institute of Child Development and Education, University of Amsterdam, Amsterdam, The Netherlands

**Keywords:** PTSD, Psychotherapy, Adolescent, Resting-state fMRI, Machine learning, Clinical outcome

## Abstract

Randomized controlled trials have shown efficacy of trauma-focused psychotherapies in youth with posttraumatic stress disorder (PTSD). However, response varies considerably among individuals. Currently, no biomarkers are available to assist clinicians in identifying youth who are most likely to benefit from treatment. In this study, we investigated whether resting-state functional magnetic resonance imaging (rs-fMRI) could distinguish between responders and non-responders on the group- and individual patient level. Pre-treatment rs-fMRI was recorded in 40 youth (ages 8–17 years) with (partial) PTSD before trauma-focused psychotherapy. Change in symptom severity from pre- to post-treatment was assessed using the Clinician-Administered PTSD scale for Children and Adolescents to divide participants into responders (≥30% symptom reduction) and non-responders. Functional networks were identified using *meta*-independent component analysis. Group-differences within- and between-network connectivity between responders and non-responders were tested using permutation testing. Individual predictions were made using multivariate, cross-validated support vector machine classification. A network centered on the bilateral superior temporal gyrus predicted treatment response for individual patients with 76% accuracy (p_FWE_ = 0.02, 87% sensitivity, 65% specificity, area-under-receiver-operator-curve of 0.82). Functional connectivity between the frontoparietal and sensorimotor network was significantly stronger in non-responders (*t* = 5.35, p_FWE_ = 0.01) on the group-level. Within-network connectivity was not significantly different between groups. This study provides proof-of-concept evidence for the feasibility to predict trauma-focused psychotherapy response in youth with PTSD at an individual-level. Future studies are required to test if larger cohorts could increase accuracy and to test further generalizability of the prediction models.

## Highlights

• ML and rs-fMRI have shown promise in predicting treatment-response in adults with PTSD.
• Currently, no biomarkers for treatment-response are available in youth with PTSD.
• FC between the FPN and SMN was stronger in treatment non-responders on the group-level.
• A network within the bilateral STG predicted response for individual youth with 76% accuracy.
• Future studies should test generalizability of these findings and test if larger cohorts increase accuracy.

## 1 Introduction

Posttraumatic stress disorder (PTSD) is a common mental health disorder that develops in approximately 16% of youth exposed to traumatic events (Alisic et al., 2014). Youth with PTSD are troubled by frequent re-experiencing of the traumatic event, persistent avoidance, hyperarousal and negative alterations in cognition and mood (American Psychiatric Association, 2013). These symptoms can interfere with social functioning and school performance, have a negative effect on quality of life (Carrion et al., 2002) and are a crucial factor in shaping the vulnerability to depression and suicidality later in life (Molnar et al., 2001). Randomized controlled trials (RCTs) have demonstrated the efficacy of trauma-focused psychotherapies in youth with PTSD (Morina et al., 2016), but response varies considerably among individuals, with 30–50% of youth not benefiting sufficiently (Diehle et al., 2015, Morina et al., 2016). Different pre-treatment clinical and demographic factors have been associated with trauma-focused psychotherapy outcome, but none have shown to reliably predict treatment response (Goldbeck et al., 2016). This underlines the need for the identification of reliable (bio)markers of treatment response which could assist clinicians to optimize treatment allocation and improve clinical outcome.

Previous studies have shown that adult PTSD is characterized by functional alterations in brain regions which are key nodes in multiple large-scale brain networks, including the insula and medial prefrontal cortex (Wang et al., 2016). The insula is part of the salience network (SN) that is responsible for detecting and orienting to salient stimuli (Menon, 2011), and the medial prefrontal cortex is part of the default mode network (DMN) that is associated with internally focused thought as well as autobiographical memory (Menon, 2011). Results from studies examining large-scale network connectivity in youth with PTSD have not always corresponded with results obtained in adults (Weems et al., 2019). Patriat and colleagues, for instance, found that pediatric PTSD is characterized by increased connectivity within the DMN, contrasting the finding of decreased connectivity within the DMN in adults (Patriat et al., 2016). This could be related to considerable reorganization of large-scale brain networks throughout childhood and adolescence (Weems et al., 2019). Developmental change in large-scale brain organization is characterized by stronger within-network connectivity and more efficient between-network connectivity, with a trend towards segregation (decrease in connectivity strength) between regions in close proximity and integration (increase in connectivity strength) between anatomically distant regions (Menon, 2013). These developmental processes provide a potential explanation for the contrasting findings between youth and adults with PTSD and emphasize the need for studies on large-scale brain networks specifically performed in youth with PTSD.

Few studies have investigated the relationship between large-scale brain network connectivity and treatment-response. In adults, neuroimaging studies have observed pre-treatment differences between responders and non-responders to trauma-focused psychotherapy (Duval et al., 2020, Fonzo et al., 2017a, Fonzo et al., 2021, Korgaonkar et al., 2020, Zantvoord et al., 2013, Zhutovsky et al., 2019). Findings from these studies suggest that activity and connectivity within regions and networks involved in working memory as well as emotional processing and modulation differed between responders and non-responders at baseline (Duval et al., 2020, Zhutovsky et al., 2019) and could be adaptively attenuated with successful trauma-focused psychotherapy (Fonzo et al., 2021). A study in adolescent girls reported greater pre-treatment bilateral amygdala activation during emotion processing in treatment responders and differences in large-scale brain network connectivity (Cisler et al., 2016). These studies provide initial evidence for group-differences in pre-treatment brain activity and connectivity between treatment responders and non-responders.

The studies reported above used univariate analysis to detect group-differences. However, this does not provide information for individual patients and may not generalize to new data (Arbabshirani et al., 2017), which is necessary to allow clinicians to inform patients and to assist in clinical decision making. Predictions for individual patients can be made using multivariate supervised machine learning (ML) analysis which directly assesses generalization to new patients by means of cross-validation. Several studies have utilized ML methods and resting-state functional magnetic resonance imaging (rs-fMRI) to predict treatment-response in adults with PTSD, with accuracies ranging between 71 and 90% (Etkin et al., 2019, Korgaonkar et al., 2020, Zhutovsky et al., 2019). However, no studies are available that have investigated the utility of ML and rs-fMRI to predict treatment-response in youth with PTSD. Therefore, we collected pre-treatment rs-fMRI data of 40 youth with PTSD/partial-PTSD (age 8–17) to predict treatment response on the group- and individual-level.

## 2 Materials and methods

### 2.1 Participants

Our initial sample consisted of 61 participants (39 girls/22 boys) diagnosed with PTSD or partial PTSD. Participants entered trauma-focused psychotherapy as part of an RCT comparing trauma-focused cognitive behavioral therapy (TF-CBT) and eye movement desensitization and reprocessing (EMDR) (Diehle et al., 2015). Of these, 50 completed treatment as well as pre- and post-treatment assessment (see flow diagram in Figure S1). After data quality control 40 participants (26 girls/14 boys) were included in the final analysis. All participants were Dutch speaking, and 8–17 years old. Gender categories were based on the personal identification of participants' own gender. Participants were recruited between June 2011 and September 2018 at the outpatient child psycho-trauma center of the department of child and adolescent psychiatry, de Bascule in Amsterdam, The Netherlands. Youth were referred by child welfare services, physicians or general practitioners. Diagnoses for PTSD or partial PTSD were established clinically by an experienced child and adolescent psychiatrist or psychologists according to the DSM-IV-TR criteria using joint child and caregiver reports on individual symptoms on the Clinician-Administered PTSD Scale for Children and Adolescents (CAPS-CA) semi-structured interview (Nader et al., 1996) and the caregiver reports from the PTSD scale of the Anxiety Disorders Interview Schedule – Parent Version (ADIS-P) (Verlinden et al., 2014). A symptom was established as present, if either child or caregiver reported its presence. Partial PTSD was defined as either fulfilling two of the three PTSD symptom clusters or having one symptom present in each of the three symptom clusters (Stein et al., 1997). Furthermore, participants were required to have a CAPS-CA total score indicating at least mild PTSD symptom severity (>20 points). Exclusion criteria were: acute suicidality, IQ < 70, pregnancy, neurological disorders or serious medical illnesses or meeting the criteria of the following diagnosis: psychotic disorders, substance-use disorder or pervasive developmental disorder. If participants were taking psychotropic or central nervous-active medication, medication was required to be stable for at least three weeks before and during trauma-focused psychotherapy. In our sample one participant was taking sertraline and two methylphenidate. In accordance with procedures approved by the Institutional Review Board of the Amsterdam University Medical Center and the declaration of Helsinki, written informed consent was obtained from all parents or legal guardians. Written informed consent from youth aged 12 years and older and assent from youth aged 11 and younger, was also obtained from the youth themselves. All participants received a monetary incentive for participation (€5 for each assessments).

### 2.2 Trauma-focused psychotherapy

Participants were randomly assigned to weekly protocolized sessions for a total of 8 weeks of either TF-CBT or EMDR. The data reported here were obtained as part of a larger study on the efficacy of TF-CBT and EMDR. Treatment was delivered by experienced trauma therapists who were trained in TF-CBT and EMDR before study initiation. Supervision by TF-CBT and EMDR experts was provided throughout the study. Treatment protocols, training and supervision of therapists, as well as treatment fidelity have been described in detail previously (Zantvoord et al., 2019).

Trained psychologists administered the CAPS-CA and the PTSD scale of the ADIS-P to measure PTSD symptoms before and after treatment. Caregiver reports on the ADIS-P were used to complement child reports and clinical observation. The Dutch Revised Child Anxiety and Depression Scale (RCADS(-P)) questionnaires was administered to assess depressive and anxiety symptoms (Chorpita et al., 2000). Symptom change was calculated by subtracting the pre-treatment from the post-treatment CAPS-CA total score. We used ≥30% reduction of CAPS-CA total score as response criterion for clinically meaningful improvement (Zantvoord et al., 2021).

The distribution of baseline clinical, trauma and demographic characteristics across responders and non-responders was examined using X*^2^*-tests, independent sample *t*-tests or Mann-Whitney tests as appropriate. Paired sample *t*-test were used to examine pre- to post-treatment symptom change. Statistical analyses were performed using SPSS version 26 (SPSS Inc., Chicago IL, USA).

### 2.3 Imaging data acquisition

High-resolution T1 and rs-fMRI data were acquired using a 3T Philips Achieva scanner (Philips Healthcare, Best, The Netherlands) equipped with a SENSE eight-channel receiver head coil. For each participant, a T1-weighted structural MRI image was acquired with the following parameters: TE: 3.527 ms, TR: 9 ms, slice thickness: 1 mm, 170 slices, flip angle: 8◦ and image matrix 256 × 256 that covert the entire brain. 200 blood oxygen level dependent rs-fMRI scans were acquired with a repetition time of 2.3s and a voxel size of 2.3x2.3x3mm^3^. For rs-fMRI, participants were instructed to remain still with their eyes closed.

### 2.4 Imaging data preprocessing

All (f)MRI preprocessing was performed utilizing a singularity image container running fMRIPrep (v1.5.3^3^).

#### 2.4.1 Structural data preprocessing

Structural MR images were corrected for intensity non-uniformity and brain-extracted using the ANTs toolbox (v2.2.0^4^). Brain tissue segmentation of cerebrospinal fluid (CSF), white-matter (WM), and gray-matter (GM) was performed on the brain-extracted T1w images using FSL FAST (v5.0.9). Volume-based spatial normalization to MNI space (MNI152NLin6Asym) was performed through nonlinear symmetric normalization with ANTs.

#### 2.4.2 Functional data preprocessing

Preprocessing of rs-fMRI data followed the standard procedure implemented in fMRIPrep involving generation of a reference volume, co-registration to the T1w scan, motion correction (before any spatiotemporal filtering) and normalization to MNI space in one step using a combination of all spatial transformations (see Supplementary Materials for details). Normalizations and co-registrations were assessed visually and four PTSD patients were excluded due to poor normalization quality. We excluded five additional participants with high spikes of motion identified from visual inspection of plots of the realignment parameters (volume-to-volume changes >2mm). Therefore, after quality control of the structural MRI and rs-fMRI data the final sample included 40 patients. These remaining participants did not differ in overall motion levels according to their framewise displacement (Power et al., 2014) (see Table 1). Data were spatially smoothed with an isotropic, Gaussian kernel of 6mm^3^ full-width-at-half-maximum. To further address motion contamination, we applied ICA-AROMA (Pruim et al., 2015) (in MNI space) to remove additional motion sources from the data. Data was then resampled to 4mm^3^ to speed-up additional procedures. We addressed further structured noise present in the data by regressing out average WM and CSF signals using masks calculated in T1w space, transformed to rs-fMRI space. We combined this regression step with highpass filtering by a discrete cosine set with 128s cut-off. To avoid reintroducing already removed nuisance signal into the data by applying a sequential pipeline, both WM/CSF and cosine regressors were denoised with the previously identified ICA-AROMA regressors (Lindquist et al., 2019). As a final step the rs-fMRI data were grand-mean scaled with a factor of 10000.

**Table 1:** Subject characteristics.

	Overall (n = 40)	Responders (n = 21) ≥30% CAPS-CA	Non-responders (n = 19) <30% CAPS-CA	p-value^a^
**Sociodemographic characteristics (pre-treatment)**				
Girls (%)	65.0	57.1	73.7	0.273
Age (years; mean, SD)	12.6 (2.91)	12.5 (2.64)	12.7 (3.25)	0.820
West European Ethnicity (%)	47.5	52.4	42.1	0.413
Current educational level (%)				0.557
Elementary school	47.5	52.4	42.1	
Middle/High school lower level	7.5	9.5	5.3	
Middle/High school middle level	27.5	28.6	26.3	
Middle/High school higher level	12.5	9.5	15.8	
Vocational school	5.0	0	10.5	
Household Income (€; %)				0.622
<25000	27.5	28.6	26.3	
25000–35000	12.5	19.0	5.3	
>35000	20.0	23.8	15.8	
Weight (kg; mean, SD)	51.1 (10.94)	51.3 (12.67)	50.7 (8.46)	0.875
Current psychotropic medication (%) Smoking (%) Alcohol > 1 consumption/day (%)	7.5 7.5 0	9.5 9.5 0	5.3 5.3 0	0.609 0.702 N/A
**Imaging Data (pre-treatment)**				
Framewise displacement (mean, SD)	0.20 (0.11)	0.21 (0.11)	0.20 (0.12)	0.820
**Trauma characteristics (pre-treatment)**				
Index trauma (%) Sexual abuse Domestic violence Community violence Accidents/Medical Other	32.5 12.5 25.0 12.5 17.5	28.6 14.3 23.8 14.3 19.0	36.8 10.5 26.3 10.5 15.8	0.971
Repeated trauma exposure (%)	57.5	61.9	52.6	0.554
Age at index trauma (years; mean, SD)	9.9 (3.89)	10.0 (3.43)	9.9 (4.42)	0.824
Time since index trauma (years; mean, SD)	2.8 (2.52)	2.7 (2.00)	2.9 (3.03)	0.773
**Clinical characteristics (pre-treatment)**				
CAPS-CA (mean, SD)^b^ Total Re-experiencing Avoidance Hyperarousal	56.1 (23.25) 17.8 (10.37) 21.5 (10.02) 17.8 (8.96)	55.5 (23.95) 16.7 (10.04) 22.8 (9.62) 16.8 (9.49)	56.8 (23.09) 18.9 (10.92) 20.1 (10.54) 18.8 (8.48)	0.856 0.532 0.422 0.515
Full PTSD diagnosis (%)	82.5	85.7	78.9	0.574
RCADS (mean, SD)^b^ MDD GAD OCD PD SAD SP	12.0 (6.08) 7.2 (4.30) 6.8 (3.35) 8.4 (6.25) 6.1 (4.24) 12.2 (6.76)	11.7 (6.11) 8.4 (4.56) 7.3 (3.84) 9.2 (6.65) 7.4 (3.91) 13.3 (7.28)	12.5 (6.29) 5.6 (3.48) 6.2 (2.59) 7.4 (5.81) 4.3 (4.12) 10.8 (6.00)	0.729 0.089 0.407 0.469 0.**048** 0.339
**Administered Psychotherapies**				
TF-CBT/EMDR	24/16	11/10	13/6	0.301
**Clinical characteristics (post-treatment)**				
CAPS-CA (mean, SD)^b^ Total Re-experiencing Avoidance Hyperarousal	38.0 (25.70) 10.6 (10.17) 12.1 (9.14) 12.2 (9.14)	22.3 (19.58) 5.4 (16.93) 9.4 (15.27) 6.4 (6.38)	55.2 (20.14) 16.9 (10.66) 15.3 (7.52) 19.2 (6.81)	**<0.001** **0.001** 0.062 **<0.001**

Abbreviations: CAPS-CA, Clinician-Administered PTSD Scale for Children and Adolescents; RCADS, Revised Child Anxiety and Depression Scale; MDD, major depressive disorder; GAD, general anxiety disorder; OCD, obsessive compulsive disorder; PD, panic disorder; SAD, separation anxiety disorder; SP, social phobia; SD, standard deviation; TF-CBT, trauma-focused cognitive behavioral therapy; EMDR, eye movement desensitization and reprocessing.

a p-values < 0.05 shown in bold. Independent samples *t*-test for continuous and Χ^2^ tests for categorical variables between responders and non-responders.

b Ranges: CAPS-CA total, 0–139; RCADS MDD, 0–30; RCADS GAD, 0–18; RCADS OCD, 0–18; RCADS PD, 0–27; RCADS SAD, 0–21; RCADS SP, 0–27.

#### 2.4.3 Identification of intrinsic connectivity networks

To identify a set of robust intrinsic connectivity networks (ICNs) we employed a *meta*-independent component analysis (ICA) (Biswal et al., 2010) utilizing FSL MELODIC (v3.15) (Beckmann and Smith, 2004). To ensure that the identification of ICNs was independent from their use in the analysis, which may introduce a positive bias (Poldrack et al., 2019), we included rs-fMRI data of 17 trauma-exposed controls (TEC) who did not differ in age, gender, or motion from the included patients (see Supplementary Materials for further details). The number of components was fixed to 70 as it has been successful in the identification of treatment-related PTSD biomarkers for veterans in our previous study and in addition has been shown to be repeatable able to well separate signal sources and be optimal in detecting disease-related group-level differences (Abou-Elseoud et al., 2010, Abou Elseoud et al., 2011, Zhutovsky et al., 2019). To identify ICNs, we employed a semi-automatic approach (Cerliani et al., 2015) which led to the inclusion of 48 ICNs (see Supplementary Materials). Both ICNs and excluded components are shown in Figures S2 and S3, respectively.

To reconstruct individual-level representations of the group-level ICNs and their time-courses we applied group-information guided ICA (GIG-ICA) to the preprocessed data of the PTSD patients (Du and Fan, 2013). GIG-ICA computes a spatially constrained individual-level ICA which estimates individual ICNs which are maximally spatially correlated with a group-map. This procedure is repeated for each group ICN and each participant, generating a set of individual-level ICN representations and their corresponding time-courses. GIG-ICA has been shown to outperform conventional reconstruction methods like dual regression in identifying reliable biomarkers for psychiatric disorders and to produce spatially independent components (Du and Fan, 2013, Salman et al., 2019). GIG-ICA was applied utilizing MATLAB code (R2018b, The Mathworks, Natick, MA) distributed with the GroupICA toolbox (v4.0b^5^).

To investigate between-ICN connectivity we applied the FSLnets toolbox (v0.6.3^6^) to the individual-level ICN time-courses estimated via GIG-ICA. We estimated full- and partial correlation matrices between all identified ICNs and converted all correlation coefficients to z-scores for further analyses. Full-correlation matrices were estimated using Pearson correlation while partial-correlation matrices were calculated from regularized Ridge regressions (rho = 0.1) as is the default in the FSLnets package.

### 2.5 Group-level analyses

We tested for group-differences across ICNs (within-ICN connectivity) between responders and non-responders using permutation testing implemented in PALM (a117^7^). We included demeaned age, gender and pre-treatment CAPS-CA total scores as covariates-of-no-interest into a general linear model (GLM). Familywise error (FWE) correction of p-values across the whole-brain, 48 ICNs and two-sided tests of the threshold-free-cluster-enhancement (TFCE) statistic (Smith and Nichols, 2009) was performed using synchronized permutations (n = 10000) of the maximum statistic.

The same procedure, involving permutation testing (n = 10000), and the same covariates-of-no-interest was utilized to investigate group-difference in between-ICN connectivity across responders and non-responders. The FWE-correction of p-values of the *t*-statistic was performed across all connections, two-sided contrasts and the full- and partial correlation matrices utilizing the maximum statistic. Alpha was set to 0.05 in both analyses.

### 2.6 Individual-level analyses

To investigate whether within- or between-ICN connectivity could predict treatment-response for the individual patient, we applied multivariate, cross-validated linear-kernel support vector classifiers (SVM) (Cortes and Vapnik, 1995) to our data. For that we considered every ICN (n = 48) and their connectivity profiles (full- and partial correlation matrices, n = 2) separately, resulting in 50 separate multivariate classification analyses. We divided our data into 5-folds (each fold containing 20% of the data) ensuring (approximate) balance of responders and non-responders per fold. Data of 4-folds was used as training set for rescaling all features to =1 to 1 range and fitting the SVM. The fifth fold served as the test set and we calculated balanced accuracy (average between sensitivity and specificity), area-under-the-receiver-operator-curve (AUC), sensitivity (of identifying responders), specificity (of identifying non-responders) and negative/positive predictive value (NPV/PPV) as performance measures of the trained SVM classifier. We repeated the procedure five times, each time retraining the classifier and utilizing a different fold as the test set. Finally, to ensure a reliable average measure of classification performance we repeated the random division of the data across the five folds 50 times and repeated the entire analysis, yielding a 50-times-repeated-5-fold cross-validation procedure (Varoquaux et al., 2017). In the end, we averaged the performance measures across the 250 test set evaluations, providing a set of measures estimating the generalizability of our classifier to new data.

To assess statistically whether the estimated average accuracies provided better-than-chance performance and to correct for the total number of classifications performed (48 ICNs + 2 correlations matrices = 50), we used synchronized permutation tests (n = 2000, see Supplementary Materials). Alpha was set to 0.05.

We also assessed which features were important for the classification by calculating p-values for each weight of the SVM using a novel permutation-based procedure (Gaonkar et al., 2015) (see Supplementary Materials). The p-values were computed after the classifier was applied to the entire data set and are intended for visualization purposes only.

All individual-level analyses were implemented in the Python programming language (v3.8.2) utilizing the scikit-learn ML toolbox (v0.22.1).

## 3 Results

### 3.1 Demographic and clinical characteristics

A summary of participant characteristics is shown in Table 1. Treatment responders and non-responders did not differ in demographic, trauma and clinical characteristics at baseline apart from separation anxiety symptoms which were (marginally significantly) higher in responders (p = 0.048). Based on joint child (CAPS-CA) and caregiver (ADIS-P) reports 82.5% of all participants met the full DSM-IV diagnostic criteria for PTSD at baseline, the remaining 17.5% met criteria for partial PTSD. The average baseline CAPS-CA score was 56.13 (SD = 23.25), which is indicative of moderately severe PTSD. The most common index trauma was sexual abuse, followed by community violence, accidents and domestic violence. 57.5% of participants were exposed to multiple-event trauma. Average age at trauma exposure was M = 9.95 years, SD = 3.89 (range 2–16) and average time since trauma was M = 2.82 years, SD = 2.52 (range 0–10).

### 3.2 Changes in psychopathology

Treatment completers and non-completers did not differ in baseline sociodemographic, trauma or clinical characteristics. Across the completer sample, we found significant reductions in CAPS-CA total score (*t*(39) = 5.65, p < 0.001, Cohen’s effect size (d) = 0.89), re-experiencing (*t*(39) = 4.39, p < 0.001, d = 0.71), avoidance (*t*(39) = 4.10, p < 0.001, d = 0.68) and hyperarousal clusters (*t*(39) = 2.935, p = 0.006, d = 0.55. Twenty-one fulfilled the criterion for treatment response (≥30% PTSD symptom reduction on CAPS-CA), and nineteen were non-responders.

### 3.3 Resting-state fMRI

#### 3.3.1 Group-level analyses

##### 3.3.1.1 Within-network analyses

There were no group-differences surviving FWE-correction between responders and non-responders for any of the 48 ICNs. Because the number of investigated components was large, requiring stringent correction for multiple comparisons, we also provide the results of the analyses when FWE-correction was only applied for each network separately (see Figure S4. Within-network connectivity of two ICNs (left frotoparietal network (FPN) and a visual ICN) was increased in responders over non-responders in this exploratory analysis.

##### 3.3.1.2 Between-network analyses

**Fig. 1:**
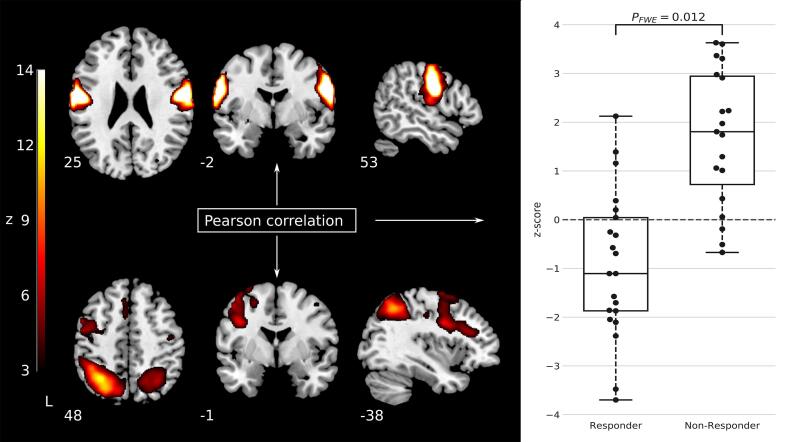
Stronger Fisher r-to-z transformed Pearson correlation between a sensorimotor network and the (predominantly) left frontoparietal network was observed for non-responders over responders. Boxplots show median and interquartile range of the distribution of responders/non-responders. The dots show the individual z-transformed correlation values of the individual patients. It is important to note that the individual correlation values shown in the boxplot cannot be directly used to infer the performance in the classification analysis as this would constitute ‘double dipping’ (Kriegeskorte et al., 2009).

Between-network analyses showed a significantly larger Pearson correlation between the (predominantly) left FPN and a sensorimotor network in non-responders over responders (*t* = 5.35, p_FWE_ = 0.012, Fig. 1.

#### 3.3.2 Individual-level analyses

**Fig. 2:**
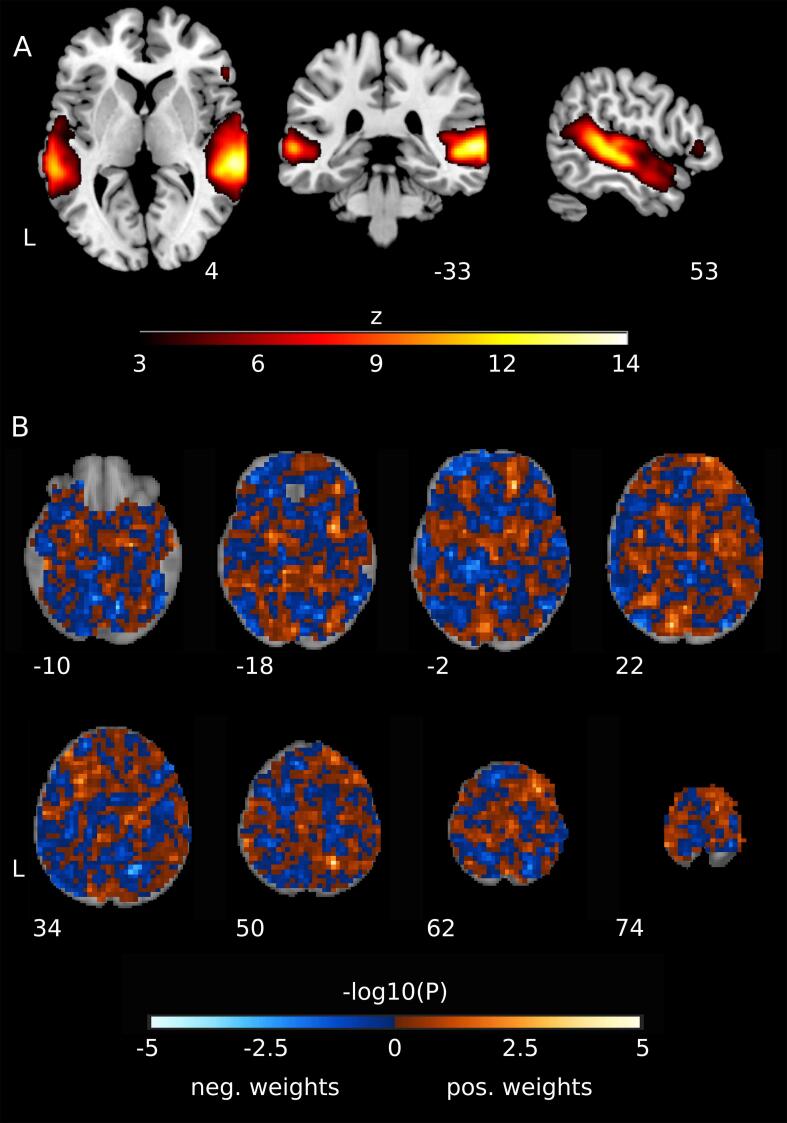
A. A network centered on the bilateral superior temporal gyrus which provided the best performance during the multivariate classification of responders and non-responders. The network was part of the 70 networks computed by means of *meta*-ICA on the (independent) HC sample. B. p-values of the individual voxel weights of the SVM estimated using the margin-aware statistic and analytical approximation of the null-distribution (Gaonkar et al., 2015) for classification using the individual-level representation of the group network in A. p-values are shown unthresholded as the analysis is multivariate and therefore all voxels – and not only the most significant ones – always contribute to the classification task.

**Fig. 3:**
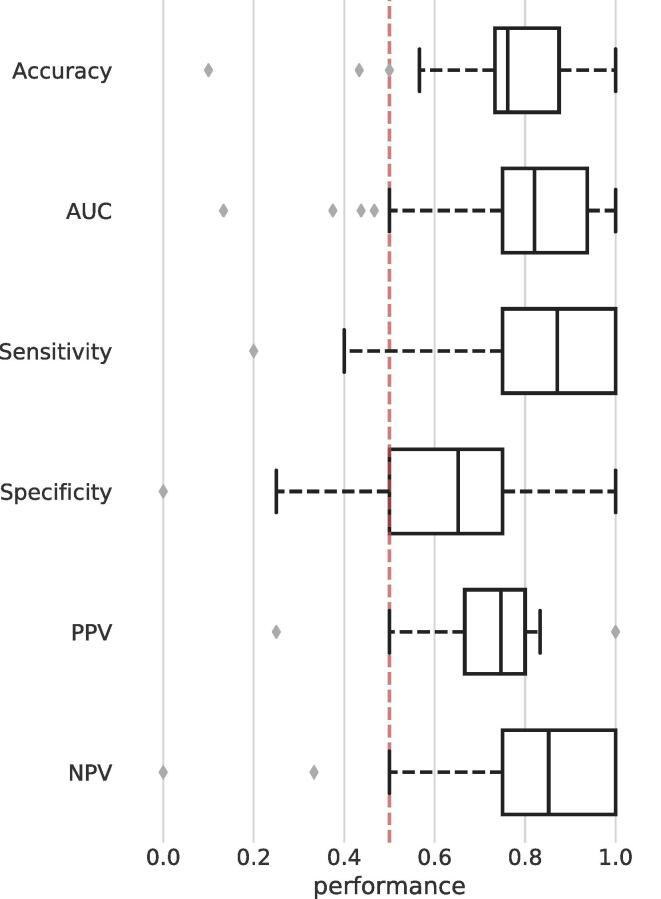
Cross-validated performance estimates of the best performing network during classification (Fig. 2). Boxplots show the mean and interquartile range (IQR) of the individual performance distributions. The mean instead of the median is shown because it was also used and reported as final performance measure of the network. The red dotted line indicates approximate chance-level. However, statistically, deviation from chance-level and FWE-correction were estimated through synchronized permutations. (For interpretation of the references to colour in this figure legend, the reader is referred to the web version of this article.)

SVMs trained on data from an ICN centered on the bilateral superior temporal gyrus (STG) provided an average cross-validated accuracy of 76.17% (SD = 12.58%, p_FWE_ = 0.018, Fig. 2A and Fig. 3). The network achieved an AUC of 0.82 (SD = 0.16), with a sensitivity of 87.14% (SD = 16.56%) and a specificity of 65.20% (SD = 21.44%). The PPV/NPV was 0.75/0.85 (SD = 0.14/0.19). To explore whether the test accuracy was comparable for TF-CBT and EMDR, we also tested the same model for this ICN for the different treatments separately. The cross-validated performance for the TF-CBT subgroup showed a balanced accuracy of 76.16% (AUC: 0.82, sensitivity: 85.97%, specificity: 64.3%). For the EMDR subgroup a balanced accuracy of 78.56% (AUC: 0.83, sensitivity: 83.43, specificity: 52.0%) was observed. We also investigated how the usage of a different validation approach (leave-one-out cross-validation) would influence the performance estimates of the best performing network: we observed an accuracy of 69.42%, AUC of 0.77, sensitivity of 80.95%, and specificity of 57.89%. However, given that there are theoretical and empirical reasons for why leave-one-out cross-validation is not recommended – especially in the case of small sample sizes – the reported results from this validation scheme should not be the focus of this study (Flint et al., 2021, Poldrack et al., 2019). To illustrate that there is a significant advantage in utilizing multivariate instead of univariate models we performed an experiment in which we selected the best separating voxel (according to a *t*-test performed on the training set) of the above network and trained and tested our linear SVM models using only this one voxel. The obtained performance dropped significantly to an averaged accuracy of 50.99%, AUC of 0.54, sensitivity of 53.12% and specificity of 48.87%. No other network showed classification accuracies exceeding chance-level when FWE-correction was applied.

P-values corresponding to the voxel-weights of the SVM classifier when trained on data of all patients of the STG ICN can be seen in Fig. 2 (B), showing a diffuse whole-brain pattern required to successfully perform the classification.

## 4 Discussion

In this study we investigated the possibility of using pre-treatment rs-fMRI data as a biomarker to predict trauma-focused psychotherapy response in youth with (partial) PTSD. We examined prediction both on the group- and individual-level. In our study, a network centered on the bilateral STG could distinguish between responders and non-responders on the individual-level, with an accuracy of 76.2%. We further found increased connectivity between the left FPN and a sensorimotor network in non-responders on the group-level. To our knowledge this is the first study to examine the prediction of individual treatment-response using rs-fMRI data in youth with PTSD. Together our results provide a first proof-of-concept for the utility of rs-fMRI as a biomarker for treatment-response in youth with PTSD.

Our findings indicate increased pre-treatment connectivity between the left FPN and sensorimotor network in trauma-focused psychotherapy non-responders. The FPN is highly integrated with other brain networks and has a comprehensive role in attention, working memory and decision making by flexibly interacting with other brain networks (Menon, 2011). Abnormal recruitment of other brain networks into the FPN is linked with deficits in these cognitive processes and has been associated with multiple psychiatric disorders (Menon, 2011). More specifically, increased connectivity between the FPN and a sensorimotor network has been found in youth with autism (ASD) and attention-deficit/hyperactivity disorder (ADHD) (Cerliani et al., 2015). While speculative at this point, abnormal recruitment of the sensorimotor network into the FPN in non-responders might be related to deficient cognitive processes resulting in suboptimal engagement in trauma-focused psychotherapy and poor treatment response. To test this hypothesis, future research could address functional connectivity patterns of the FPN together with neurocognitive tests before and after treatment and use repeated transcranial magnetic stimulation to directly influence FPN connectivity (Etkin et al., 2019). Such an approach could eventually delineate clinical relevance and might identify promising targets for non-invasive stimulation-based interventions (Fonzo et al., 2017b).

Our group-level analysis did not identify networks which were previously found to distinguish between trauma-focused psychotherapy responders and non-responders in adult PTSD. More specifically, we did not find group differences in connectivity between the SN and DMN which was found to differentiate veterans who responded to prolonged exposure (PE) therapy compared to non-responders (Sheynin et al., 2020). Additionally, previous findings of lower ventral attention network (VAN) connectivity and increased connectivity in the frontal pole in adults with poor trauma-focused psychotherapy response were also not replicated (Etkin et al., 2019) (Zhutovsky et al., 2019). One possible explanation for these divergent findings could be that most previous studies used a region of interest approach focusing on predefined networks contrary to our whole-brain analysis. In addition, different types of psychotherapies and clinical as well as trauma characteristics could have accounted for these differences. And finally, developmental processes could have contributed as large-scale brain networks undergo considerable reorganization throughout childhood and adolescence (Menon, 2013).

The ICN yielding significant classification performance was centered on the STG. A growing number of studies have shown structural and functional abnormalities in the STG in PTSD patients (Engdahl et al., 2010, Lanius et al., 2002, Lindauer et al., 2008). Based on electrical stimulation of the area, Engdahl and colleagues (Engdahl et al., 2010) have suggested that STG abnormalities may be associated with re-experiencing symptoms. Others have suggested a relationship between STG abnormalities and dissociative symptoms in PTSD patients (Lanius et al., 2002). Interestingly, we have previously shown a positive correlation between STG activation and trauma-focused psychotherapy response in adults with PTSD (Lindauer et al., 2008).

Previous studies utilizing ML methods, however, did not identify network connectivity of the STG as an accurate predictor of treatment response. In adults treated with PE, Etkin and colleagues, found a classification accuracy of >85%, using a combination of pre-treatment rs-fMRI connectivity within the VAN and delayed recall performance in a verbal memory task (Etkin et al., 2019). In another study, pre-treatment functional connectivity within- and between- the default mode, dorsal attention, cingulo-opercular, salience, and central executive network during task-free fMRI predicted response to TF-CBT with an accuracy of 71.4% (Korgaonkar et al., 2020). Finally, we have previously shown the feasibility of the same approach as outlined here to predict response to trauma-focused therapy in veterans with PTSD with 81.4% accuracy (Zhutovsky et al., 2019), with an ICN centered on the pre-supplementary motor area providing the best predictive accuracy.

At present, it remains unclear why our findings on classification accuracy differ from findings in adult PTSD. Studies in adults have reported different networks/functional connectivity estimates than identified here and have found classification accuracies which mostly exceeded accuracy found in the current study. One possibility is that, with inclusion of both PTSD and partial PTSD patients, clinical heterogeneity increased, resulting in lower classification accuracy. Another possibility is that neurodevelopmental trajectories add to heterogeneity and might reduce classification accuracy, as previous studies in youth with PTSD using rs-fMRI have shown neurodevelopmental effects on network connectivity and we included youth with a relatively wide age range. These hypotheses require further investigation, including longitudinal studies of youth with PTSD which develop into adulthood. While the current individual-level classification findings differ from adults, it is reassuring that the application of the same approach to treatment-response classification as reported here has been associated with significant classification accuracies in adults multiple times, even for a different psychiatric disorder (van Waarde et al., 2015, Zhutovsky et al., 2019).

There is a difference between the findings observed on the group- and on the individual-level. While there was no difference in within-network connectivity for any ICN between responders and non-responders on the group-level, there was a network significantly predictive on the individual-level. The opposite was true for the between-network connectivity. These discrepancies can be explained by the fact that a significant p-value in group-comparisons does not have to imply the ability to distinguish between patients on the individual-level because of low effect sizes of the difference (Arbabshirani et al., 2017). In addition, both analyses have different goals and therefore can identify different ICNs: group-level analyses focus on determining localized average differences between groups while individual-level analyses utilize all multivariate data to determine a model which provides the highest prediction (Bzdok and Ioannidis, 2019). This clearly marks the importance of performing individual-level prediction studies as these may improve clinical decision making in the future and may lead to independent results from group-level studies.

Although classification accuracy exceeded chance-level performance, it still falls below the APA proposed threshold for clinical applicability of biomarkers (First et al., 2018). The suggested combination of >80% sensitivity, specificity, and PPV is useful as guidance for research, but clinical utility should preferably be based on cost-benefit analyses (Pepe et al., 2016). As the current clinical standard is to offer trauma-focused psychotherapy to all youth with PTSD, a biomarker which reliably identifies non-responders could aid clinical decision making. This would correspond to a classifier with high specificity, but also reasonably high sensitivity to prevent classifying all patients as non-responders. If a-priory chances of treatment non-response are high, clinicians together with patients and their caregivers, could decide to abstain from initiating trauma-focused psychotherapy and search for alternative treatments with higher chances of success. This may help to prevent the unnecessary burden of failed treatment trials.

Several limitations of this study should be noted. First, the sample size in the current study is low. This has an impact on the certainty of the estimated performance of the individual-level analysis. Cross-validation can lead to high variance in performance estimates if applied to studies with low sample sizes (Varoquaux, 2018). To increase the confidence in the presented results, we followed best-practices for the field (Poldrack et al., 2019), utilizing a permutation test corrected for multiple comparisons to provide a valid statistical control of the observed performance (Varoquaux, 2018). However, only with larger sample sizes can these problems be fully addressed and therefore the current study can only be regarded as a first step for further individual-level prediction studies in youth with PTSD. Larger sample sizes at the same time may increase clinical heterogeneity, limiting classification performance as well (Arbabshirani et al., 2017). Second, although the majority (82.5%) of included youth had a full PTSD diagnosis, the remaining 17.5% had a partial PTSD diagnosis. Including youth with partial PTSD increased clinical heterogeneity. Increased clinical heterogeneity might have lowered overall treatment response due to a floor effect and might have lowered prediction accuracy. However, by including youth with partial PTSD, our sample better reflects the real-life clinical setting, which adds to the ecological validity of our findings. Third, youth were randomized to receive either TF-CBT or EMDR, and both treatment conditions were collapsed for the current analysis. Due to limited power it was not feasible to examine differences between treatment responders and non-responders separately for both treatments or examine specific predictors for each treatment separately. However, we exploratively investigated the selective performance of the STG ICN for the different treatment groups in our sample which showed a similar performance to the classifier applied to the combined group. This indicates that the network is predictive of treatment-response in both treatment groups. Importantly, efficacy of both treatments has been shown to be comparable in an RCT with considerable sample overlap with the current study (Diehle et al., 2015). In addition, three of the 40 included patients were taking psychotropic medication. While one of the inclusion criteria of the study was that medication usage had to be stable for at least three weeks before and during trauma-focused psychotherapy, this could have influenced our results. However, excluding more patients would have limited our sample size even more which is why we chose not to do it. Furthermore, the relatively wide age range (8–17) of the included patients might have influenced the results of the current study as functional networks may be represented differently across development of youth (Menon, 2013). Finally, our study had substantial drop-out, as 18% of randomized patients were lost to follow-up. Although such dropout rates reflect routine clinical practice and treatment completers and non-completers did not differ on baseline characteristics, there is a possibility that drop-out could have influenced our findings through attrition bias.

### 4.1 Conclusions

The present study demonstrates that increased resting-state connectivity between the FPN and a sensorimotor network can distinguish trauma-focused psychotherapy responders from non-responders on the group-level. Future studies could examine if these network patterns are potential targets for (non-invasive) neuromodulation interventions to reduce PTSD symptoms in afflicted youth. We further show that resting-state connectivity patterns in a network centered on the bilateral STG are capable of predicting trauma-focused psychotherapy response in youth with PTSD. These proof-of-concept findings emphasize the feasibility of combining ML analysis and rs-fMRI to identify predictive biomarkers for treatment response. However, before translation to clinical practice can commence, future research should aim to test the robustness and generalizability of these findings in larger independent cohorts.

#### CRediT authorship contribution statement

**Paul Zhutovsky:** Methodology, Software, Validation, Visualization, Writing – original draft. **Jasper B. Zantvoord:** Conceptualization, Methodology, Investigation, Writing – original draft. **Judith B.M. Ensink:** Investigation, Writing – review & editing. **Rosanne op den Kelder:** Investigation, Writing – review & editing. **Ramon J.L. Lindauer:** Conceptualization, Supervision, Funding acquisition, Writing – review & editing. **Guido A. van Wingen:** Methodology, Supervision, Funding acquisition, Writing – review & editing.

## Declaration of Competing Interest

The authors declare that they have no known competing financial interests or personal relationships that could have appeared to influence the work reported in this paper.

## Footnotes

3 https://fmriprep.org/en/1.5.3/

4 https://stnava.github.io/ANTs

5 https://trendscenter.org/software/gift

6 https://fsl.fmrib.ox.ac.uk/fsl/fslwiki/FSLNets

7 https://fsl.fmrib.ox.ac.uk/fsl/fslwiki/PALM
